# Lecture XLII

**Published:** 1871-12

**Authors:** N. S. Davis


### CHICAGO MEDICAL EXAMINER:

N. S. DAVIS, M. D., EDITOR.

F. H. DAVIS, M. D., ASSISTANT EDITOR.

VOL. XII. NOV. AND DEC., 1871. NOS. 11 & 12.

### ORIGINAL CONTRIBUTIONS.

### LECTURE XLII.

Erysipelas—Sporadic and Epidemic—Being one of the Lectures in the Regular Course on Practical Medicine in the Chicago Medical College, for the Session of 1871–2.

BY N. S. DAVIS, M D., and Prof. Reported by L. S.

GENTLEMEN: There is another disease that was appended to the class of affections which has occupied our attention for the last few days, but which, as we explained at the time, presented so many points of difference from the other members of the class, that it was not strictly correct to call it an eruptive fever. That disease is Erysipelas. It is one of the few diseases that seem to form a connecting link between the departments of Practical Medicine and Surgery. When it occurs in connection with wounds, injuries, or surgical operations, it is called *traumatic erysipelas*, and the consideration of this form shall leave entirely to my colleague in the Chair of Principles and Practice of Surgery.

The disease also sometimes attacks the woman in child-bed, giving rise to one of the most fatal forms of puerperal fever. But you will be duly instructed in reference to this by my colleagues in the departments of Obstetrics and Gynæcology. Erysipelas, however, appears both sporadically and epidemically independent of wounds or injuries, and equally independent of the child-bed state; and when thus occurring it is as fully within the domain of practical medicine as pneumonia or typhoid fever. In this aspect it is not a disease of frequent occurence, yet the busy practitioner meets with cases of it almost every season. Occasionally it assumes the form of an epidemic and presents every grade of severity. When it occurs sporadically, that is, without any prevalent epidemic tendency, it is generally mild and rarely proves fatal. On the other hand, in some of the epidemics that have occurred in this country, it has assumed such a degree of severity as to destroy life in three or four days.

One of the most noted epidemics that have come within my own observation prevailed from 1841 to 1846, in different parts of the country. It appeared first, if we remember correctly, in some of the eastern states and extended westward through the central part of New York, not by a continuous line of communication, but more or less irregularly, and after appearing in the western states continuing to recur in different localities until 1846. When the meat packing business first became extensive in this city, if I remember right, it was in 1863, several large slaughtering houses were erected on the south branch of the river, and early in the autumn the blood and offal was turned into that sluggish stream in such quantity that it became literally red with blood to its outlet in the lake. The rapid decomposition of this quantity of animal matter soon killed all the fish in the river, and impregnated the atmosphere with effluvia to such an extent as to be offensive to the nostrils in almost every part of the city. In less than four weeks erysipelas began to prevail, and within three months several hundred cases occured. More than seventy cases came under my own care. The disease was so clearly traceable to the emenations from the river, that my colleague, Prof. E. Andrews, constructed the following map, showing the localities of its prevalence.

During the prevalence of an epidemic of erysipelas there is generally more than the usual prevalence of puerperal fever or peritonitis. It has been observed that a large proportion of the women who are confined during the prevalence of an epidemic in the circle of its influence, are attacked with puerperal or child-bed fever; and this is usually accompanied by a large ratio of mortality.

The connection of erysipelas of the epidemic character with cerebro-spinal meningitis has been noticed in several localities. We have had an unusual prevalence of erysipelas, at least three times since I have been a resident of Chicago; one of these was the one I alluded to as caused by the putrid emanations from the Chicago River. It was a marked epidemic in which a large number of people were attacked; but it was not accompanied by a large ratio of mortality.

The disease as it occurs sporadically, is generally ushered in by a chill often so moderate as to attract but little attention and immediately followed by a moderate degree of fever. But there are no symptoms to distinguish it from the onset of any other febrile affection, except that there is usually a slight stiffness and soreness in the fauces, sometimes so positive as at once to attract attention, and other times the patient will hardly allude to it, unless it be inquired after particularly. At the same time that the fever comes up active enough to give rise to heat of skin, general feelings of uneasiness, headache, and pains in the back and limbs, there is some degree of thirst with a little whitish or yellowish white coat upon the tongue. At the end of the first day, or during the second, there is usually a feeling of soreness in the flesh and stiffness directly over the wing of the nose, and on the ridge of the cheek or under the tip of the ear. This is true of a majority of instances; but there are instances in which this particular feeling of soreness in the flesh, as if the part had been bruised, is not enough to attract attention. The same feeling of soreness may be in other parts, as the upper extremities or the trunk of the body. Occasionally it is the nates and sometimes the scrotum.

In about 24 hours from the time this soreness is felt in any given locality there will be manifested symptoms of inflammation on the skin. This inflammation will be of a deep red color with some tumefaction and hardness showing a little extravasation or exudation into the subcutaneous tissue. The elevated red spot has a perfectly defined margin. In this respect it differs from most other inflammations. The margin is more abrupt and better defined ; it is not shaded off so insensibly that you cannot really tell where the redness begins as in ordinary inflammation.

In the moderate cases of erysipelas the soreness in the throat that has been previously felt during the two or three days of premonitory fever disappears as soon as the local inflammation appears on the surface. The eruption or tumefaction generally begins on the cheek near the wing of the nose but there are many instances in which it makes its appearance first at the tip of the ear. Whichever place it begins it spreads steadily and usually with considerable rapidity laterally, and in all directions except directly downward toward the lower lip or the chin. It extends laterally and upwards more and more upon the cheek so that in about three days from the time it appeared, in ordinary cases of sporadic erysipelas it will have spread over the whole of that side of the face including the eyelids and crossed over the nose so as to reach about midway over the other cheek. It always crosses over the bridge of the nose, never on the lips below it. I do not know why, but I have watched erysipelas a good many years and never knew a case to cross underneath the nostril on the lips but it invariably goes over the bridge of the nose.

In mild cases it will continue to spread steadily about one week, when it will occupy the entire face (except the chin and lips) including the ears and mastoid spaces. Many cases of the milder class will stop there, and after remaining stationary for one or two days will decline rapidly leaving the patient convalescent in from nine to twelve days from the commencement of the attack.

Oftentimes in these mild cases there will appear about the third day after the inflammation on the surface more or less vesication. These are not distinct vesications of uniform character, like varicella or herpes, but they will vary in size from the circumference of a very small pea to the diameter of one or two inches constituting large blebs or simple elevations of cuticle, the spaces being generally filled with transparent serum so that they look precisely like blisters produced by the application of cantharides. These vesicles in some mild cases are entirely absent. In cases of moderate severity they are quite extensive and often run together.

In the class of cases a little more severe, the disease does not stop when it occupies the face, but keeps steadily on until it extends over the whole scalp, ears, and back of the neck. There are many cases where it recedes from the point first attacked in proportion as it advances to new parts. For instance if it commences on one side and spreads laterally, by the time it comes to occupy the opposite side and pass over the eyelids and upon the forehead, the first cheek invaded will have the disease declining so that the swelling has partly disappeared from it; the vesicles will be drying up and the cuticle exfoliating; and by the time it invades the top of the head, it will be passing from the face, which will become in a great measure free. Often where the patient was attacked eight or ten days before, the face will be nearly well, and the disease occupying the back side of the head and back of the neck and ears and mastoid spaces.

But in the most severe class of cases it does not decline at the point first attacked as it advances to new parts, but extends more rapidly and the tumefaction will be so great that in the course of five or six days from the onset of the disease the eyes will be closed completely and the features will not be rcognizaable, the swelling will have extended over the whole head, face ears and mastoid spaces, and the back of the neck and the face will present large vesications. In such instances there is apt to be more or less delirium; the pulse increased to 100 or 120 beats in a minute, and is tolerably firm under the finger; the skin over the whole body is hot; the patient will have a severe grade of general fever. His tongue and fauces will be more or less dry, especially a strip along the middle of the tongue; frequently the fauces will be sore, so that there will be some pain in the act of deglutition, and the glands occasionally become involved in the swelling behind the angle of the jaws, so that there is more or less difficulty in opening the mouth. These are the severer class of cases occurring sporadically.

When the disease has prevailed as an epidemic, especially in the epidemic that occurred from the year 1841 to 1846, in some localities it assumed a still greater degree of severity. The patients would be attacked with a chill, followed immediately or accompanied we might say, by soreness and stiffness, in the fauces; and followed by a rapid development of general fever. In twenty-four hours the erysipelatous redness would make its appearance upon the cheek near the wing of the nose and spread rapidly, swelling the features largely, and in four or five days it would have extended over the face, head, and neck. The disease would continue in the throat, tumefying the tonsils and adjacent parts. At the end of six or seven days the tongue would become swollen so as to fill the mouth, making both breathing and deglutition difficult and noisy.

In some cases the tongue becomes so much swollen as to fill the mouth, compelling the patient to keep it open; allowing the tongue to become covered with a dark crust. It was this swollen and black appearance that gave the disease very generally the name of "black tongue."

Cases of this degree of severity were accompanied early by delirium, sometimes excited delirium, and in from four to six days, in some, the throat would be so completely filled up that suffocation and death ensued.

It is possible that some of the cases involved more or less inflammation of the base of the brain. A great many cases, however, in that epidemic were of a milder character. One of the cases that occurred in the neighborhood where I was practicing, but not under my care, was a young woman. The disease attacked the cheek first, but extended over the face, head, and down upon the back of the neck, over the shoulders, and finally over the trunk of the body, so that the entire head, neck, and trunk to the waist anteriorly, and hips posteriorly were occupied by the disease, accompanied by great tumefaction, intense redness, and numerous large vesications. This patient died. At the same time this patient was sick a case occurred in my own practice. The patient was a man who was not in the habit of being very abstemious in his drinking or eating and was of a sanguine temperament. He was attacked with the disease violently. It was ushered in by a chill and soreness in the fauces ; it next attacked the face extensively and spread so rapidly that in forty-eight hours it covered the whole face and head, the swelling completely closing the eyes and going as far back as the ccipital and mastoid regions. He became wildly delirious, the pulse full and firm under the finger, and the skin hot and dry. I opened a vein and bled him freely, taking not less than thirty ounces of blood from the arm, and put a blister about an inch wide around his neck. The idea was prevalent at that time that we could put a stop to the spread of the disease by applying nitrate of silver so as to cauterize the cuticle or by removing it by blistering. I put a blister around the neck, and kept him on a strictly antiphlogistic regimen for five or six days subsequently ; and whether the treatment had any effect or not the man got well.

The disease did not go below the blistered surface on the neck. This does not prove, however, that erysipelas will not cross a blistered surface, because I have learned from my own experience that it will, and I have also seen it cross directly over the surface where it has been thoroughly cauterized with nitrate of silver. In this case the treatment appeared to give a decided check to the disease. The bleeding I have no doubt was decidedly beneficial.

In some cases where the disease has prevailed in its epidemic form and the face, neck and throat became severely affected, these parts are left œdematous after the beginning of convalescence. The patient being much debilitated, sometimes the aplastic or serous infiltration has extended into the tissues about the base of the epiglottis and between the vocal cords. This causes a moderate amount of tumefaction taking the name of *œdema of the glottis*, which affects the patient precisely like the severest form of croup and is sometimes the cause of suffocating the patient in the course of a few hours.

It was in consequence of some of these cases of œdema of the glottis following erysipelas and typhus fever that Dr. Gurdon Buck first attempted to scarify the epiglottis so as to allow of the escape of the exuded material; and he succeeded in some instances in the New York Hospital, in apparently rescuing patients from death. If any of you have access to the transactions of the American Medical Association, you will find a paper from him on this subject with a very excellent colored plate representing the œdema of those parts, and a description of the mode of proceeding in this surgical process of relief. I do not recollect what year he published the article.

As the disease prevailed here during the epidemic to which I have previously alluded in the year 1863, we had a considerable number of cases that were badly managed during the early stage of the disease. Some occurred in private practice, and others were brought into the hospital in the advanced stage of the disease, in which there was exhibited what I have not seen in any other epidemic, namely an extensive suppurative process set up especially in the parts most thoroughly tumefied during the progress of the disease. I recollect three cases that were brought into the hospital in which the disease had already spread over the whole face and head, completely occupying the surface except the chin; the swelling was very great; the eyes were completely closed, and large vesications existed on different portions of the surface, looking purplish from the intermixture of blood with the serum in the vesicles. About the time when the inflammation and swelling should begin to abate, suppuration took place deeply in the tissues of the eyelids, and in two cases the eyeballs were completely encircled except at the angles by abscesses, destroying the cutaneous tissue and suppurating deeply into the socket, making it look for a time as though the eyeballs were going to be dissected out.

Still these patients recovered, and when the ulcers had filled up with granulations, as they did slowly, and cicatrized there was less difiguration than I had feared. There was a moderate degree of ectropion or turning out of the lower eyelids from the contraction of the cicatrices.

We have another form of erysipelas called phlegmonous, which generally attacks the extremities though it may attack different parts of the body. The disease is ushered in by more or less rigor, and the fever is generally more of the typhoid grade. The redness, when it appears upon the surface is darker or more purplish; the margin of the tumefactions not so well defined; the swelling permeates the tissues much more deeply and spreads less rapidly than ordinary erysipelas upon the surface. This variety of erysipelas generally spreads only a short space from the place of the first beginning, but speedily involves partial gangrene of the areolar tissue and diffuse suppuration. In from five to seven days we get an obscure sense of fluctuation but the abscesses have no distinct boundaries and do not tend to point at any particular place. On the contrary the matter burrows extensively between the muscles. As soon as you find a feeling of obscure fluctuation in the part, if you make a free incision, you will give exit to a considerable quantity of ill-formed pus, together with more or less strips or shreds of areolar tissue, undissolved but completely dead and seperated from the living tissue. Often these shreds blocks up the incisions and temporarily obstruct the flow of matter, requiring them to be pulled out with a bent probe or forceps.

This form of erysipelas not unfrequently occurs in hospitals where the air is bad, in military camps, and sometimes in private practice where the patients are occupying low, wet and ill-ventilated dwellings; it is also liable to attack persons in the stage of recovery from typhoid and typhus fevers. Something resembling phlegmonous erysipelas will sometimes attack the parotid region during the progress of recovery from continued fever. I have a patient now who was one of those unfortunate ones burned out by the recent great fire, previously accustomed to good living, but suddenly stripped of house and home, she was compelled to accept of such accommodations as she could get. She was attacked in a short time by severe typhus fever. It ran a pretty severe course of about three weeks, when she was fairly convalescent, so that I considered her safe, and omitted my visits for two or three days, when I was hastily recalled. I found her suffering from a diffuse and rapidly increasing swelling in both parotid regions. There was a blush of purplish redness upon the surface, and the parts were hard and inelastic to the touch. By putting the patient on the tincture of chloride of iron internally and covering the swollen parts carefully with cloths saturated in an infusion of aconite leaves and muriate ammonia, the progress of the swelling was checked, and in the four or five days that have elapsed, it has nearly disappeared. But in a great many of these cases you will not be so fortunate as to check the progress of the swelling. The jaws will become completely blocked; deglutition will become difficult; the patient already debilitated by previous disease, either sinks from complete exhaustion; or lingering for a week or ten days, the areolar tissue involved in the swelling becomes destroyed partly by suppuration and partly by gangrene. If early and free incisions are made, discharging the pus and shreds of dead tissue, a slow and tedious recovery may be the result. But all these cases involve danger to the life of the patient.

TREATMENT:—Believing erysipelas to be a specific disease, caused by an animal poison, either imbibed with the air and water from without, or engendered by some imperfection of the disintegrating processes within; I cannot fully agree with the majority of writers on practical medicine, who state in general terms that it must be treated on the same principles as the continued fevers.* Speaking of the epidemic occurring from 1841 to 1846, Dr. Flint says: "tonic remedies, alimentation, and alcoholic stimulants, in other, supporting measures of treatment, were indicated." Very similar views are expressed by

---

* See Flint's Practice of Medicine, pp. 850–52.

Drs. Bennett, Aitkins, and Tanner. The two latter admit that the disease is sometimes sthenic when measures of a more antiphlogistic character may be indicated.

None of these writers speak of the tincture of chloride of iron, except as a simple tonic of more or less value in asthenic cases. My experience with erysipelas, both in its epidemic and sporadic form, has satisfied me that, while it is a specific disease and amenable to the influence of certain remedial agents, it occurs in very different conditions of the system.

A very few cases will present, during the first few days, that fulness and firmness of pulse ; heat and tension of the skin ; intensity of headache with some degree of delirium, that will not only justify, but render a prompt venesection, highly beneficial.

One case of this kind we have already mentioned.

In a much larger number of cases, in which the abstraction of blood is not necessary, there is much benefit derived from giving at the commencement five grains of calomel with five of bi-carbonate of soda, and if they do not cause a movement of the bowels in four or five hours, aiding them by a saline laxative. Immediately after the proper evacuations, I commence the use of some one of the remedies that are regarded as more directly curative in their influence over the disease. Abundant opportunities for observation have satisfied me that the tincture of chloride of iron, and the sulphites of soda and lime, are capable of exerting as specific an influence over the progress of idiopathic erysipelas, as quinine is over the paroxysms of an intermittent fever. And in a large majority of the cases, as they are presented to the practitioner, no preparatory treatment by evacuents or alteratives, is necessary, before commencing the use of this class of remedies. If the tincture of chloride of iron is relied on, it should be given to adults in doses of from 20 to 30 drops, in half a wine-glassful of sweetened water, every two or three hours, according to the severity of the case. After the first forty-eight hours, the interval between the doses may be increased to three or four hours, and continued at that rate until the redness and swelling have disappeared, and convalescence is fully established. Under this treatment the disease generally ceases to extend its boundaries after the third day, and by the seventh convalesence is fairly begun. In some cases a mild laxative may be needed two or three times during the progress of the case; and in other instances the bowels will become relaxed in the advanced stage, needing the exhibition of five or six grains of Dover's Powder each night.

In a few cases the tincture of chloride of iron has disturbed the stomach sufficiently to cause its rejection to vomiting. When this happens, the most reliable substitute is the sulphite of soda, in doses of from 10 to 20 grains dissolved in a small wineglassful of water, and repeated every two, three or four hours. In the epidemic of 1863, I tested the efficiency of the sulphites of soda and lime, in several very severe cases, and was well satisfied with the results. During the same season six of the most severe cases admitted into the Mercy Hospital, were complicated with a typhoid grade of dysentery. To these I gave twenty grain doses of sulphite of lime mixed in a table spoonful of sweet milk, every four hours, and ten drops each of oil of turpentine and tincture of opium in a teaspoonful of an emulsion of gum-arabic and sugar, half way between the doses of the sulphite. The uniform result was a speedy improvement in the symptoms and an early convalescence.

When erysipelas occurs in a malarious district, the fever accompanying it exhibits, generally, more or less of a periodical character. In such cases the same remedies should be given, with the addition of anti-periodic doses of sulphate of quinine at each remission, in the early stage; and smaller doses may be combined with the iron in the later stage. During the first few years of my residence in this city, before the present system of sewerage and water-supply had been adopted, I met with several cases of erysipelas thus associated with malarious fever, and found the use of quinine an indispensable part of the treatment.

During the first three or four days in ordinary cases of this disease, the diet should consist of farinacious articles and milk, being careful not to have the latter given immediately after the doses of tincture of chloride of iron. Subsequently, especially in the more asthenic cases, wheat-flour and milk porridge, beef-tea, and other animal broths, should be administered in moderate quantities and at short intervals, sufficient to afford a fair degree of support without overloading the stomach. When the treatment of the disease has been such as now recommended from the first two or three days after the commencement of the attack, I have never known a dangerous degree of debility to supervene during any part of its progress.

But in some cases that had been neglected or unnecessarily purged during the first five or six days, at a more advanced period, strongly marked typhus symptoms have supervened, such as muttering delirium ; sub-sultus ; a quick, soft and weak pulse ; and imperfect control over the discharges. At the same time the erysipelatous surface becomes purplish color, with more or less œdematous infiltration into the adjacent tissues.

In the few cases of this character that have come under my observation, I have given either moderate doses of carbonate of ammonia, camphor and quinine ; or strychnine and nitric-acid, alternately with the doses of tincture of chloride of iron, with decided benefit. Most writers have recommended the use of wine and other alcholic remedies in the treatment of erysipelas. But regarding these agents as neither tonic nor supporting to the vital processes, I have never used them in this form of disease. That their omission has not been detrimental to my patients, is proved by the fact that during the whole period of my practice, only one patient has died from the disease who came under my care before the end of the first week after the commencement of the attack. That case was an infant only three weeks old. The disease commenced on the nates and spread successively over almost every inch of the cutaneous surface. The stomach was so irritable that very little of either medicine or nourishment was retained, and it died about the end of the first week. It is gratifying to find my position in relation to the use of alcoholic remedies in the treatment of this disease, fully sustained by the experience of Dr. Hartshorne, as given in his work entitled "Essentials of Practical Medicine." *

In phlegmonous erysipelas, in addition to the internal treatment, free incisions should be made into the swollen tissues, as soon as evidences of suppuration are discernable. I have thus far said nothing in regard to external local applications in the treatment of erysipelatous inflammation. When I entered upon the practice of medicine, much emphasis was placed on the value of such applications. Nitrate of silver, iodine, solutions of sulphate of copper, iron, zinc, and acetate of lead, all had their strenuous advocates. It required but a few years of observation at the bed-side, to satisfy me that no local applications were capable of exerting any controlling influence over the progress of the disease, and in most instances, they only added to the annoyance of the patient. Hence, for the last fifteen years I have used no local applications except in such cases as the patients were anxious to have something that would temporarily lessen the heat and burning of the surface. I have allowed such to keep the surface moistened with a mixture of three parts of glycerine and one of sub-acetas plumbi, which answers the purpose as well as anything that I have been able to devise. It has been claimed that erysipelas is liable to metastasis, that is, suddenly to recede from the surface and attack internal organs, as the brain, lungs, stomach, peritoneum, etc. No such cases have come under my observation. But should such be presented, no better rule can be given for their management, than to treat them on the same principles that you would a similar grade of inflammation in the same organs from any other cause.

---

### BROMIDE POTASSIUM IN URÆMIC CONVULSIONS.

By EDWIN A. CARPENTER, M. D., Baileyville, Ill.

DEAR EXAMINER: I have recently had a case in practice that may interest some of the multitude of your readers, more particularly, perhaps, the junior members thereof. I report it

---

* See Essentials of Practical Medicine, pp. 321-2.

